# Diagnostic Accuracy of Molecular Imaging Techniques for Detecting Prostate Cancer: A Systematic Review

**DOI:** 10.3390/diagnostics14131315

**Published:** 2024-06-21

**Authors:** Abdullah Fahad A. Alshamrani

**Affiliations:** Department of Diagnostic Radiology Technology, College of Applied Medical Sciences, Taibah University, Madinah 42353, Saudi Arabia; ashamrani@taibahu.edu.sa

**Keywords:** molecular imaging, prostate cancer, detection, PET/CT, PET/MRI, PSMA

## Abstract

Molecular imaging modalities show valuable non-invasive techniques capable of precisely and selectively addressing molecular markers associated with prostate cancer (PCa). This systematic review provides an overview of imaging markers utilized in positron emission tomography (PET) methods, specifically focusing on the pathways and mediators involved in PCa. This systematic review aims to evaluate and analyse existing literature on the diagnostic accuracy of molecular imaging techniques for detecting PCa. The PubMed, EBSCO, ScienceDirect, and Web of Science databases were searched, identifying 32 studies that reported molecular imaging modalities for detecting PCa. Numerous imaging modalities and radiotracers were used to detect PCa, including ^68^Ga-prostate-specific membrane antigen (PSMA) PET/computed tomography (CT), ^68^Ga-PSMA-11 PET/magnetic resonance imaging (MRI), ^18^F-PSMA-1007 PET/CT, ^18^F-DCFPyL PET/MRI, ^18^F-choline PET/MRI, and ^18^F-fluoroethylcholine PET/MRI. Across 11 studies, radiolabelled ^68^Ga-PSMA PET/CT imaging had a pooled sensitivity of 80 (95% confidence interval [CI]: 35–93), specificity of 90 (95% CI: 71–98), and accuracy of 86 (95% CI: 64–96). The PSMA-ligand ^68^Ga-PET/CT showed good diagnostic performance and appears promising for detecting and staging PCa.

## 1. Introduction

Prostate cancer (PCa) is a common form of cancer affecting men worldwide [1]. Early PCa detection is crucial for effective treatment and improved patient outcomes. In recent years, molecular imaging techniques have emerged as promising tools for detecting PCa, its recurrence, and its metastasis [2]. These techniques offer the potential for increased accuracy and timely detection, allowing for more targeted and personalised treatments. Conventional imaging methods such as computed tomography (CT) and magnetic resonance imaging (MRI) currently have limited sensitivity for detecting early-stage or low-grade PCa [3]. Consequently, molecular imaging techniques targeting specific PCa biomarkers, such as the prostate-specific membrane antigen (PSMA) and androgen receptor (AR), have recently gained significant attention [4,5,6,7,8]. These techniques offer a distinct advantage by enabling a thorough and precise delineation, along with quantification, of biological processes at the cellular level in vivo.

Recent developments in imaging tracers and exceptionally targeted probes have elevated molecular imaging into a field of considerable interest [9,10]. There are several types of molecular imaging techniques, including conventional planar (2D) scintigraphy, single-photon emission computed tomography (SPECT), positron emission tomography (PET), and hybrid modalities. The SPECT and PET imaging techniques use gamma cameras and different isotopes (positrons) for imaging, respectively [11,12]. These radionuclides can be coupled with suitable ligands possessing available binding sites [13]. Various molecular imaging modalities, such as PET and MRI, and radiotracers have historically been used to examine patients with known PCa [14]. Combining techniques in multimodality probes allows the limitations of using only one type of modality to be overcome, providing increased diagnostic accuracy in detecting PCa lesions than single modalities such as CT, MRI, and bone scintigraphy [8]. These imaging techniques have emerged as accurate and precise tools for PCa staging and restaging [15,16,17,18].

There are several promising molecular imaging techniques for detecting PCa. One is PSMA PET/CT. PSMA is a protein that is overexpressed on the surface of PCa cells. PSMA PET/CT uses a radioactive tracer that binds to PSMA, allowing clinicians to visualise the cancer cells in the body [19,20]. ^68^Ga-PSMA PET/CT is now widely used at initial PCa staging in patients with high metastatic risk or biochemical recurrence [5,21]. Active clinical trials are currently evaluating many PSMA-targeting agents, including ^18^F-PSMA-1007, ^68^Ga-PSMA-617, ^68^Ga-PSMA-I&T, and ^18^F-rhPSMA-7.3 [22,23]. Another promising molecular imaging technique is fluorodeoxyglucose (FDG) PET/CT. FDG PET/CT can detect PCa by visualising the areas of the body where the cancer cells are taking up more glucose than normal cells [24]. However, FDG PET/CT is not as specific for PCa as PSMA PET/CT. Other promising molecular imaging techniques use PET radiotracers, such as ^18^F-choline, ^11^C-choline, and fluciclovine, to image patients with known PCa [15,25,26].

The diagnostic accuracy of molecular imaging in prostate cancer is influenced by many factors, such as the imaging modality, radiotracer selection, and technical parameters such as resolution and signal-to-noise ratio [7]. Patient-specific factors, tumour characteristics, and operator expertise also significantly impact outcomes. Additionally, technological advancements and comprehensive clinical context integration are crucial for precise and reliable diagnostics [27].

The diagnostic accuracy of molecular imaging techniques for detecting PCa is constantly evolving. As these techniques continue to develop, they will likely become the standard of care for diagnosing and staging PCa. Therefore, this study aims to systematically review the diagnostic accuracy of molecular imaging techniques for detecting PCa, focusing on their role and ability to accurately assess PCa.

## 2. Methods

### 2.1. Resources and Search Terms

A systematic search was performed in several databases including PubMed, EBSCO, ScienceDirect, and Web of Science for relevant articles published between 2010 and 2023. The search strategy used a combination of medical subject headings [Mesh] and keywords related to molecular imaging techniques and PCa. Several keywords (alone or in combination) were searched to find relevant articles: molecular imaging [*], prostate cancer [*], detecting [*], and accuracy [*].

### 2.2. Inclusion and Exclusion Criteria

All original human studies involving patients with PCa that used PET/MRI, PET/CT, MRI, or CT scans were included in this review. Reviews and clinical cases studies were excluded from this review.

### 2.3. Study Selection

The study selection and data extraction followed the Preferred Reporting Items for Systematic Reviews and Meta-Analyses (PRISMA) guidelines for conducting and reporting systematic reviews (Supplementary Materials) [28]. This systematic review focused on studies that investigated the accuracy of PCa detection using molecular imaging techniques, including all studies that investigated the use of PET scintigraphy for PCa diagnosis, prognosis, and therapy response assessment at different clinical stages.

**Figure 1:**
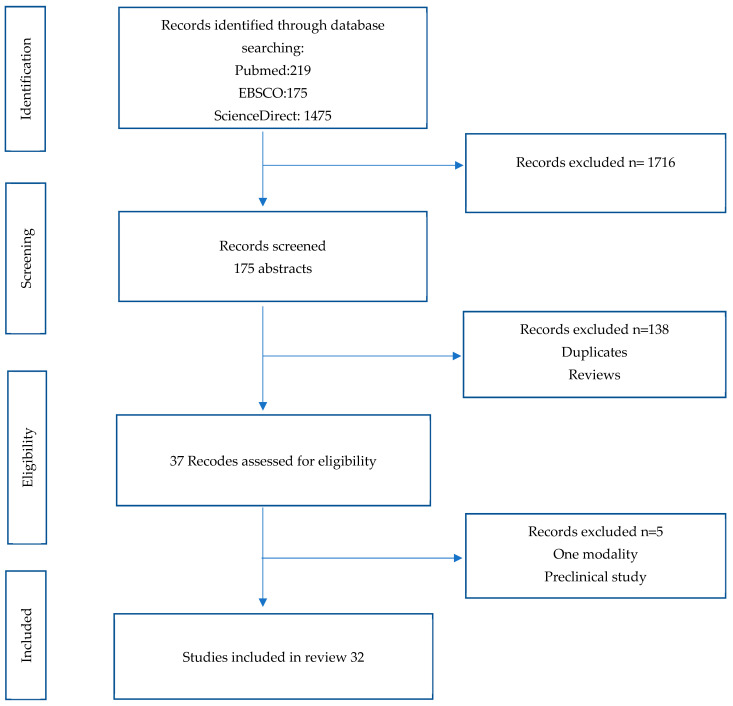
PRISM flow chart illustrates the process of identifying eligible studies for inclusion.

The initial search focused on screening the titles, abstracts, and full-text articles of potentially eligible studies for inclusion. This search was limited to articles published in English in peer-reviewed journals. An additional search was conducted to include all studies on the diagnostic accuracy of molecular imaging techniques for detecting PCa. Finally, two independent reviewers screened the titles and abstracts, and full-text articles of potentially eligible studies were assessed for inclusion. The study selection procedure is shown in a flow chart in Figure 1.

## 3. Results

The search identified 32 studies that reported molecular imaging modalities for detecting and staging PCa and its recurrence. Several imaging modalities and radiotracers were used to detect PCa, including ^68^Ga-PSMA PET/CT, ^68^Ga-PSMA-11 PET/MRI, ^18^F-PSMA-1007 PET/CT, ^18^F-DCFPyL PET/MRI, ^18^F-choline PET/MRI, and ^18^F-fluoroethylcholine (FEC) PET/MRI. Eleven studies evaluated PCa staging and accuracy with ^68^Ga-PSMA PET/CT and compared it to other modalities and radiotracers. Seven studies evaluated and compared PCa localisation with ^68^Ga-PSMA-11 PET/MRI. Six studies evaluated the accuracy of ^18^F-PSMA-1007 PET/CT and compared it to other modalities. Two studies evaluated ^18^F-2-(3-{1-carboxy-5-[(6-[^18^F]fluoro-pyridine-3-carbonyl)-amino]-pentyl}-ureido)-pentanedioic acid (DCFPyL) PET/MRI. One study evaluated ^18^F-choline PET/MRI, and another evaluated ^18^F-FEC PET/MRI. One study evaluated ^11^C-choline PET/MRI, and another evaluated ^11^C-acetate PET/MRI. Finally, two studies evaluated ^18^F-fluciclovine PET/CT. The details of these molecular imaging techniques, including their accuracy, sensitivity, specificity, positive prediction value (PPV), and negative predictive value (NPV), are summarised in Table 1. 

^68^Ga-PSMA PET/CT emerged as a prominent molecular imaging technique in the evaluated studies, with eleven investigations dedicated to assessing its efficacy in PCa staging and accuracy. The studies collectively demonstrated a consistent and notable performance of ^68^Ga-PSMA PET/CT in detecting nodal metastasis and recurrent lesions and staging primary PCa. The detection rates ranged from 64% to 96%, exhibiting its robust ability to identify primary lesions, particularly in patients with high-risk disease. Comparative analyses with other imaging modalities (MRI, CT) and radiotracers revealed that ^68^Ga-PSMA PET/CT consistently outperformed the alternatives, affirming its superiority in providing accurate and reliable information for PCa staging. Cytawa et al. demonstrated that ^68^Ga-PSMA PET/CT has a low sensitivity (35%) and high specificity (98%) for the detection of metastatic lymph nodes in patients with PCa [29]. 

Furthermore, the examination of PCa localization through ^68^Ga-PSMA-11 PET/MRI in seven studies further emphasized the versatility of this technique, showing its capability to precisely localize PCa lesions. The accuracy of this hybrid imaging modality was particularly highlighted in the assessment of PCa localization, with reported rates ranging from 74% to 99%, providing precise and comprehensive information for PCa localization. 

Finally, the efficacy of ^18^F-PSMA-1007 PET/CT was investigated in six studies, each involving a comparative analysis with alternative imaging modalities for PCa assessment. The results consistently described the robust diagnostic accuracy of ^18^F-PSMA-1007 PET/CT in detecting metastasis in recurrent and staging PCa, with reported accuracy rates ranging from 81% to 99%. Ingvar et al. reported that ^18^F-PSMA-1007 PET/CT has a low sensitivity (26%) and high specificity (96%) for the detection of lymph node metastases in PCa patients [30]. Table 2 compares the accuracy, sensitivity, and specificity of the radiolabelled ^68^Ga-PSMA PET/CT, ^68^Ga-PSMA-11 PET/MRI, and ^18^F-PSMA-1007 PET/C techniques for PCa detection.

**Table 1:** Summary of the studies that reported molecular imaging techniques for the detection of prostate cancer.

Authors, Date	Patients	Topic	Accuracy (%)	Sensitivity (%)	Specificity (%)	PPV (%)	NPV (%)
Sonni et al. 2022 [31]	74	Comparison of ^68^Ga-PSMA-11 PET/CT and mpMRI	85	77	71	-	-
Donato et al. 2020 [32]	144	^68^Ga-PSMA PET/CT for localisation of PCa.	90	91	95	-	-
Glemser et al. 2022 [16]	53	^68^Ga-PSMA-11 PET-CT, PET-MRI and MRI in recurrent PCa.	64, 67 &43	-	-	-	-
Hofman et al. 2020 [5]	150	Evaluation of ^68^Ga-PSMA PET/CT vs. CT	92	85	92	-	-
Hijazi et al. 2015 [33]	35	Evaluation of ^68^Ga-PSMA-11 PET/CT in PCa	91	94	99	89	99
Cytawa et al. 2020 [29]	82	^68^Ga-PSMA I&T PET/CT for primary staging of PCa.	93	35	98	63	95
Liu et al. 2020 [34]	31	Evaluation of ^68^Ga-PSMA PET/CT	83	93	75	77	92
Zacho et al. 2018 [35]	68	^68^Ga-PSMA PET/CT	89	80	98	89	97
Hirmas et al. 2019 [36]	21	^68^Ga-PSMA PET/CT Staging and management of patients with PCa.	85	85	-	100	-
Chandra et al. 2020 [37]	64	Accuracy of Ga-68 PSMA PET/CT	86	74	92	85	86
Chen et al. 2019 [17]	54	Combination of ^68^Ga-PSMA PET/CT and mp MRI	96	89	96	-	-
Taneja et al. 2018 [38]	35	Combined ^68^Ga-PSMA- HBED-CCPET/MRI	99	96	75	-	-
Jena et al. 2018 [39]	82	Combined ^68^Ga-PSMA-HBED-CC PET/MRI	87	90	87	-	-
Doan et al. 2023 [40]	100	Synchronous PSMA-PET/mpMRI	-	93	63	79	86
Afshar-Oromieh et al. 2014 [41]	20	Comparison of ^68^Ga-PSMA-HBED-CC PET/MRI with PET/CT	-	87	89	-	-
Al-Bayati et al. 2018 [42]	22	Evaluation of integrated ^68^Ga-PSMA-11 PET/MRI in PCa.	95	88	100	-	-
Muehlematter et al. 2019 [43]	40	Accuracy of ^68^Ga-PSMA-11 PET/MRI vs. mp MRI	74	50	94	-	-
Eiber et al. 2016 [44]	66	^68^Ga-PSMA HBED-CC PET/MRI for Localization of Primary PCa.	88	76	97	-	-
Liu et al. 2023 [45]	30	Evaluation of ^18^F-PSMA-1007 PET/CT	99	100	97	99	100
Ferraro et al. 2022 [46]	5	^18^F-PSMA-1007 PET/CT- guided biopsy in PCa.	81	65	87	-	-
Malaspina et al., 2021 [47]	31	Comparison of ^18^F-PSMA-1007 PET/CT, whole-body MRI and CT	87	87	98	83	-
Kuten et al. 2020 [48]	16	Comparison of ^18^F-PSMA-1007 PET/CT with ^68^Ga-PSMA-11	94	100	90	87	100
Mingels et al. 2022 [49]	81	Accuracy of ^18^F-PSMA-1007 PET/CT in recurrence PCa.	91	95	89	86	96
Ingvar et al. 2022 [30]	26	Accuracy of ^18^F-PSMA-1007 PET/CT		26	96	70	79
Bodar et al. 2022 [50]	30	^18^F-DCFPyL PET/MRI compared to mpMRI	83	70	89	89	90
Liu et al. 2021 [51]	52	Comparison between ^18^F-DCFPyL PET and MRI	90	93	77	95	70
Lee et al. 2017 [15]	31	Integrated ^18^F-choline PET/MRI vs. mpMRI and PET/CT	75	72	81	90	55
Hartenbach et al. 2014 [52]	38	Combined ^18^F-fluoroethylcholine (FEC) PET/MRI	82	84	80	85	78
Souvatzoglou et al. 2013 [26]	32	Comparison whole-body ^11^C-choline PET/MR with PET/CT		82	92	-	-
Polanec et al. 2017 [53]	56	MP^11^C-Acetate PET-MRI for assessment and staging PCa.	97	100	96	-	-
Zanoni et al. 2021 [54]	72	^18^F-fluciclovine PET/CT vs ^11^C-choline PET/CT	74	50	81	47	83
Odewole et al. 2016 [55]	53	Detection recurrent PCa. with^18^F-FACBC PET/CT	78	88	56	81	69

PPV, positive prediction value; NPV, negative prediction value.

## 4. Discussion

Several studies have evaluated the diagnostic accuracy of molecular imaging techniques for PCa detection with different radiotracers [15,16,17,26,29,30,31,32,33,34,35,36,37,38,39,40,41,42,43,44,45,46,47,48,49,50,51,52,53,54,55]. Various PET/CT and PET/MRI radiotracers have been investigated for diagnosing and staging metastatic PCa. The compared radiotracers include ^18^F or ^11^C-choline, ^11^C-acetate, ^18^F-fluorocyclobutane-1-carboxylic acid (^18^F-FACBC or ^18^F-fluciclovine), and those targeting PSMA. Most are radiolabelled with either Gallium-68 or Fluorine-18. ^18^F-FDG is of limited use in PCa staging due to the relatively low glycolytic activity of most PCa tumours [56].

### 4.1. Diagnostic Performance of ^68^Ga-PSMA PET/CT

PSMA is an excellent target for radionuclide PCa imaging due to its high expression in PCa cells. Recent studies have consistently demonstrated the superior diagnostic accuracy of ^68^Ga-PSMA PET/CT in various aspects of PCa detection and characterization compared to multiparametric MRI (mpMRI) and other imaging modalities. Sonni et al. 2022 found that ^68^Ga-PSMA-11 PET/CT outperformed mpMRI in detecting primary PCa, especially in high-risk cases, with detection rates of 85% and 83%, respectively [31]. Donato et al., 2020, reported higher sensitivity for PSMA-PET/CT (95%) compared to MRI (86%), without compromising specificity [32]. Glemser et al. 2020. demonstrated the superiority of ^68^Ga-PSMA-11 PET-CT and PET-MRI over MRI alone, with higher accuracy for detecting PCa relapse lesions (64% for PET-CT, 67% for PET-MRI, and 43% for MRI) [16]. Hofman et al. 2020 found that ^68^Ga-PSMA PET/CT provided superior accuracy and sensitivity for N-staging compared to combined CT and bone scan findings [5].

^68^Ga-PSMA PET/CT showed higher accuracy for detecting nodal metastasis in biochemically recurring and high-risk primary PCa. Hijazi et al., 2015, found that ^68^Ga-PSMA PET/CT had 94% sensitivity and 99% specificity [33]. Cytawa et al. 2020 reported a high accuracy of 93% and specificity of 98% for ^68^Ga-PSMA I&T PET/CT in staging primary PCa. However, they reported a low sensitivity of 35% due to the high appearance of micro-metastases [29]. Liu et al., 2020, compared the diagnostic accuracy of ^68^Ga-PSMA PET/CT and standard plus PET/CT-Ultrasound fusion targeted prostate biopsy for diagnosing clinically significant PCa. ^68^Ga-PSMA PET/CT had an accuracy of 83%, a sensitivity of 93%, and a specificity of 75% [34]. ^68^Ga-PSMA PET/CT was superior in terms of detecting metastasis in recurrent PCa compared to diffusion-weighted MRI, with a diagnostic accuracy of 89%, a sensitivity of 80%, and a specificity of 98% [35]. Moreover, ^68^Ga-PSMA PET/CT significantly impacted the initial staging and management plans for high-risk PCa patients, outperforming CT, MRI, and bone scans in terms of diagnostic accuracy [36]. Chandra et al., 2020, highlighted the high diagnostic accuracy of prebiopsy ^68^Ga-PSMA PET/CT in differentiating benign and malignant prostate lesions (sensitivity: 74%, specificity: 92%, overall accuracy: 86%) [37]. Finally, the combination of ^68^Ga-PSMA PET/CT and mpMRI demonstrated a higher diagnostic accuracy than either modality alone for detecting clinically significant PCa, with a sensitivity of 89%, specificity of 96%, and overall accuracy of 96% [17]. 

These findings collectively support the increasing role of ^68^Ga-PSMA PET/CT as a powerful tool in the diagnosis and management of PCa. Although there are some limitations for primary lesions and T and N-staging, ^68^Ga-PSMA PET/CT is the superior method for distant metastases compared to conventional methods. 

### 4.2. Diagnostic Performance of ^68^Ga-PSMA-11 PET/MRI

In a series of studies, researchers explored the diagnostic potential of combining PET/MRI techniques with PSMA tracers for PCa imaging. Jena et al. 2018 and Taneja et al. 2018 revealed that combining ^68^Ga-PSMA-HBED-CC uptake patterns with simultaneously acquired mpMRI parameters using PET/MRI improved primary PCa diagnosis compared to using each technique alone [38,39]. Al-Bayati et al. 2018 and Doan et al. 2023 found that integrated ^68^Ga-PSMA-11 PET/MRI exhibited higher diagnostic accuracy than mpMRI alone, particularly in PCa tumours [40,42]. Afshar-Oromieh et al., 2014, demonstrated that ^68^Ga-PSMA PET/MRI outperformed PET/CT, detecting recurrent PCa more easily and accurately with lower radiation exposure. PET/CT was reported to have a sensitivity of 76% and specificity of 89%, while PET/MRI was reported to have a sensitivity of 87% and specificity of 89% [41]. Muehlematter et al. 2019 compared mpMRI and ^68^Ga-PSMA-11 PET/MRI for extracapsular extension (ECE) and seminal vesicle invasion (SVI), revealing higher specificity for ^68^Ga-PSMA-11 PET/MRI [43]. Finally, Eiber et al., 2016, found that simultaneous ^68^Ga-PSMA HBED-CC PET/MRI improved the diagnostic accuracy for localizing primary PCa compared to mpMRI or PET alone, with reported accuracy, sensitivity, and specificity of 88%, 76%, and 97%, respectively [44]. These studies collectively underscore the enhanced diagnostic capabilities of combining PSMA-targeted PET imaging with MRI for various aspects of PCa assessment. In summary, there are some limitations of PET/MRI in detecting small LNs metastases, and a limited number of studies exist to compare the performance of PET/MRI against PET/CT.

### 4.3. Diagnostic Performance of ^18^F-PSMA-1007 PET/CT

In a comprehensive exploration of ^18^F-PSMA-1007 PET/CT applications in PCa, studies consistently highlight its diagnostic superiority in various contexts. Liu et al., 2023, found that ^18^F-PSMA-1007 PET/CT outperformed ^18^F-FDG PET/CT with a high accuracy of 99%, sensitivity of 100%, and specificity of 97% in detecting primary PCa lesions [45]. Ferraro et al., 2022, demonstrated the efficacy of ^18^F-PSMA-1007 PET/CT-guided biopsy for accurate lesion sampling intraoperatively [46]. Malaspina et al., 2021, showed the superiority of ^18^F-PSMA-1007 PET/CT over whole-body MRI and CT in nodal staging, reporting an accuracy of 87%, sensitivity of 87%, and specificity of 98% [47]. Furthermore, Kuten et al., 2020, compared ^18^F-PSMA-1007 and ^68^Ga-PSMA-11 PET/CT, revealing that both identified dominant prostatic lesions, with ^18^F-PSMA-1007 demonstrating a higher sensitivity of 100%. They demonstrated that ^18^F-PSMA-1007 PET/CT could also detect additional low-grade lesions of limited clinical relevance [48]. Mingels et al., 2022, highlighted the high diagnostic accuracy of ^18^F-PSMA-1007 PET/CT in detecting biochemical recurrence (accuracy: 91%, sensitivity: 95%, specificity: 89%) [49]. However, Ingvar et al., 2022, reported lower sensitivity (26%) due to small-sized metastases, but high specificity (96%) for ^18^F-PSMA-1007 PET/CT in the primary staging of lymph node metastases in intermediate- and high-risk PCa [30]. Collectively, these studies underscore the versatility and effectiveness of ^18^F-PSMA-1007 PET/CT across various diagnostic scenarios in PCa. However, ^18^F-labelled PSAM agents have not been as extensively studied as ^68^Ga-labelled agents.

### 4.4. Diagnostic Performance of ^18^F-DCFPyL-PET/MRI

Bodar et al., 2022, directly compared ^18^F-DCFPyL-PET/MRI and mpMRI in the detection of PCa. They found that ^18^F-DCFPyL-PET/MRI had a higher PCa detection rate than mpMRI alone. The diagnostic performance of ^18^F-DCFPyL PET/MRI showed an accuracy of 83%, a sensitivity of 70%, and a specificity of 89% [50]. Moreover, Liu et al. 2021 compared the diagnostic performance of ^18^F-DCFPyL PET and mpMRI in the detection of transition zone (TZ) PCa. They found that ^18^F-DCFPyL PET had a higher diagnostic value than mp-MRI. They reported that ^18^F-DCFPyL PET had an accuracy of 90%, a sensitivity of 93%, and a specificity of 77% [51]. Finally, all studies that were found only compared ^18^F-DCFPyL-PET/MRI with mpMRI, whereas there are limited studies comparing the performance with other molecular methods.

### 4.5. Diagnostic Performance of ^18^F-Choline PET/MRI and ^18^F-FEC PET/MRI

PCa cells also show an increased uptake of ^18^F-choline. Lee et al. 2017 investigated the diagnostic value of integrated ^18^F-choline PET/MRI in comparison to mpMRI and PET/CT for detecting and localising PCa. They reported superior sensitivity, accuracy, and diagnostic value with integrated PET/MRI, emphasizing its better performance over mpMRI and PET/CT [15]. Integrated ^18^F-choline PET/MRI exhibited the highest accuracy among the assessed modalities. Hartenbach et al., 2014, found that combined ^18^F-FEC PET/MRI achieved very high sensitivity (84%), specificity (80%), and accuracy (82%) in detecting dominant malignant prostate lesions, surpassing the diagnostic capabilities of PET or MRI alone [52]. The weakness of these agents has been assessed in a limited number of studies. 

### 4.6. Diagnostic Performance of ^11^C-Choline/Acetate PET/MRI

^11^C-choline showed superiority and gained popularity in the primary staging of PCa. Souvatzoglou et al., 2013, compared integrated whole-body ^11^C-choline PET/MRI to PET/CT for detecting and localizing PCa, finding higher diagnostic accuracy with PET/MRI (sensitivity: 82%, specificity: 92%) [26]. Polanec et al., 2017, assessed multiparametric ^11^C-acetate PET/MRI for PCa staging, reporting remarkable accuracy (97%), 100% sensitivity, and 96% specificity [53]. This method provided valuable insights into molecular and metabolic processes, especially in high-risk PCa cases. The main weaknesses of these methods include the limited sensitivity for detected small lesions and the short half-life of the ^11^C agent. 

### 4.7. Diagnostic Performance of ^18^F-Fluciclovine PET/CT

^18^F-Fluciclovine, also known as FACBC, performed better in evaluating PCa and recurrent disease. Zanoni et al. 2021 recently assessed ^18^F-fluciclovine PET/CT for nodal staging in high-risk primary PCa, revealing 74% accuracy, 50% sensitivity, and 81% specificity [54]. The study showed better specificity for ^18^F-fluciclovine over ^11^C-cholin. In addition, Odewole et al. 2016 found ^18^F-FACBC PET/CT to be more effective than CT in detecting recurrent prostate cancer, with 78% accuracy, 88% sensitivity, and 56% specificity [55]. Although ^18^F-FACBC PET/CT has shown good performance in recurrent metastasis, it has no significant role in primary staging detection.

This review focused on collecting data on the diagnostic performance of various imaging modalities in patients with PCa. The current study’s data show the good diagnostic performance of PET/CT or PET/MRI with different radiopharmaceuticals, which have high accuracy in detecting metastases and provide valuable insights for diagnosing, staging, and managing PCa. Molecular imaging techniques can add clinical benefits compared to conventional imaging for detecting PCa lymph node metastasis and recurrence after radical prostatectomy [2]. The limitation of this review is the limited number of studies on the diagnostic performance of all PET/MRI tracers in PCa, which reduces the statistical power of the analysis. 

The diagnostic accuracy of molecular imaging techniques for detecting PCa has been a subject of research in recent years. Therefore, more research is needed to confirm these findings and to determine the optimal use of these techniques in clinical practice.

## 5. Conclusions

Molecular imaging techniques offer several advantages over traditional imaging techniques for PCa detection, including increased accuracy, personalised treatment, timely detection, and comprehensive assessment. PSMA-ligand PET/CT is superior to conventional methods for detecting distant metastases, mainly due to its greater accuracy. ^68^Ga-PSMA PET/CT offers an approach with moderate sensitivity and high specificity for detecting metastatic LNs and staging PCa. Furthermore, PET/MRI with different radiopharmaceuticals has shown promising accuracy and effectiveness for PCa diagnosis and management. Further research is needed to explore the diagnostic accuracy of these techniques in different patient populations and disease stages.

## Figures and Tables

**Table 2:** A summary of the accuracy, sensitivity, and specificity of radiolabelled ^68^Ga-PSMA PET/CT, ^68^Ga-PSMA-11 PET/MRI, and ^18^F-PSMA-1007 PET/CT used for prostate cancer.

Imaging Modality	Number of Studies	Accuracy (%)	Sensitivity (%)	Specificity (%)
^68^Ga-PSMA PET/CT	11	86 (64–96)	80 (35–93)	90 (71–98)
^68^Ga-PSMA-11 PET/MRI	7	88 (74–99)	82 (50–96)	86 (63–100)
^18^F-PSMA-1007 PET/CT	6	90 (81–99)	78 (26–100)	92 (87–98)

Mean (minimum–maximum).

## Data Availability

No new data were created or analyzed in this study.

## Supplementary Materials

The following supporting information can be downloaded at: https://www.mdpi.com/article/10.3390/diagnostics14131315/s1.
